# Evaluation of Kharma scale as a predictor of lower third molar extraction difficulty

**DOI:** 10.4317/medoral.22082

**Published:** 2017-10-21

**Authors:** Abdurrahman A. Al-Samman

**Affiliations:** 1 B.D.S. M.Sc. Oral and Maxillofacial Surgery. Specialist, Department of the Oral Surgery, Tikrit Specialized Dental Centre /Salahiddin Health Directorate/ Ministry of Health/ IRAQ

## Abstract

**Background:**

The Evaluation of the degree of lower third molar (L3M) extraction difficulty is extremely important for both clinicians and patients. This study aimed to evaluate the validity of a new index (Kharma scale) as a preoperative predictor index of the difficulty of surgical removal of impacted L3M.

**Material and Methods:**

Extraction difficulty of a series of 49-impacted L3M was predicted preoperatively by Kharma scale, and postoperative difficulty was assessed with a modified Parant scale.

**Results:**

The sensitivity of Kharma scale, as a predictor of difficulty, was 18.2% and the specificity was 68.4%. Likelihood ratios for the Kharma categories also indicated that the scale is of little value for predicting a difficult extraction. There was no significant association between the Kharma score and duration of operation, but high-modified Parant scores were significantly associated with longer operations.

**Conclusions:**

The proposed Kharma scale was unreliable as preoperative predictor of the L3M extraction difficulty, and both radiological and clinical information must be taken into account.

** Key words:**Kharma scale, prediction scale, lower third molar, extraction difficulty.

## Introduction

Evaluation of the degree of lower third molar (L3M) extraction difficulty is extremely important to design correct treatment strategy and to reduce the risk of complications. Therefore, having optimal scale to predict L3M extraction difficulty continues to challenge clinicians.

Several methods have been established for preoperative estimation of difficulty, but they found invalid (1-4) or of limited clinical use (2,3,5,6).

Among these scales, is the Pederson scale, which is widely cited in oral and maxillofacial surgical texts as a useful tool to predict the difficulty of extraction of L3M (1). However, diverse clinicians have questioned its performance (1,3).

Recently, Kharma *et al.* (7) proposed a new scale; which is a modification of Pederson scale; that took into account the anatomical form of the tooth roots. They claimed that this new estimating index is more reliable and accurate than Pederson scale, and significantly correlated with postoperative difficulty rated by modified Parant scale (7).

The aim of this study was to evaluate Kharma scale’s prediction accuracy.

## Material and Methods

We evaluated the surgical extractions of 49 L3M performed in patients (41male, 8 female, mean (SD) age 27.5(5.7) years) who presented to the Department of the Oral Surgery, The Left Specialized Dental Centre, Mosul, Iraq from November 2015 to October 2016. The ethics committee of the Iraqi Ministry of Health approved the study.

All operations were done by two surgeons who had six and nine years’ experience in oral and maxillofacial surgery, and according to standard protocols under local anesthesia.

Preoperatively, the surgeon predicted the difficulty of extraction from panoramic radiographs using Kharma scale. After the operation, difficulty was assessed using the modified Parant scale (MPS) ( Table 1). The duration of operation was also recorded by a stopwatch (from start of incision to final suture).

**Table 1:**
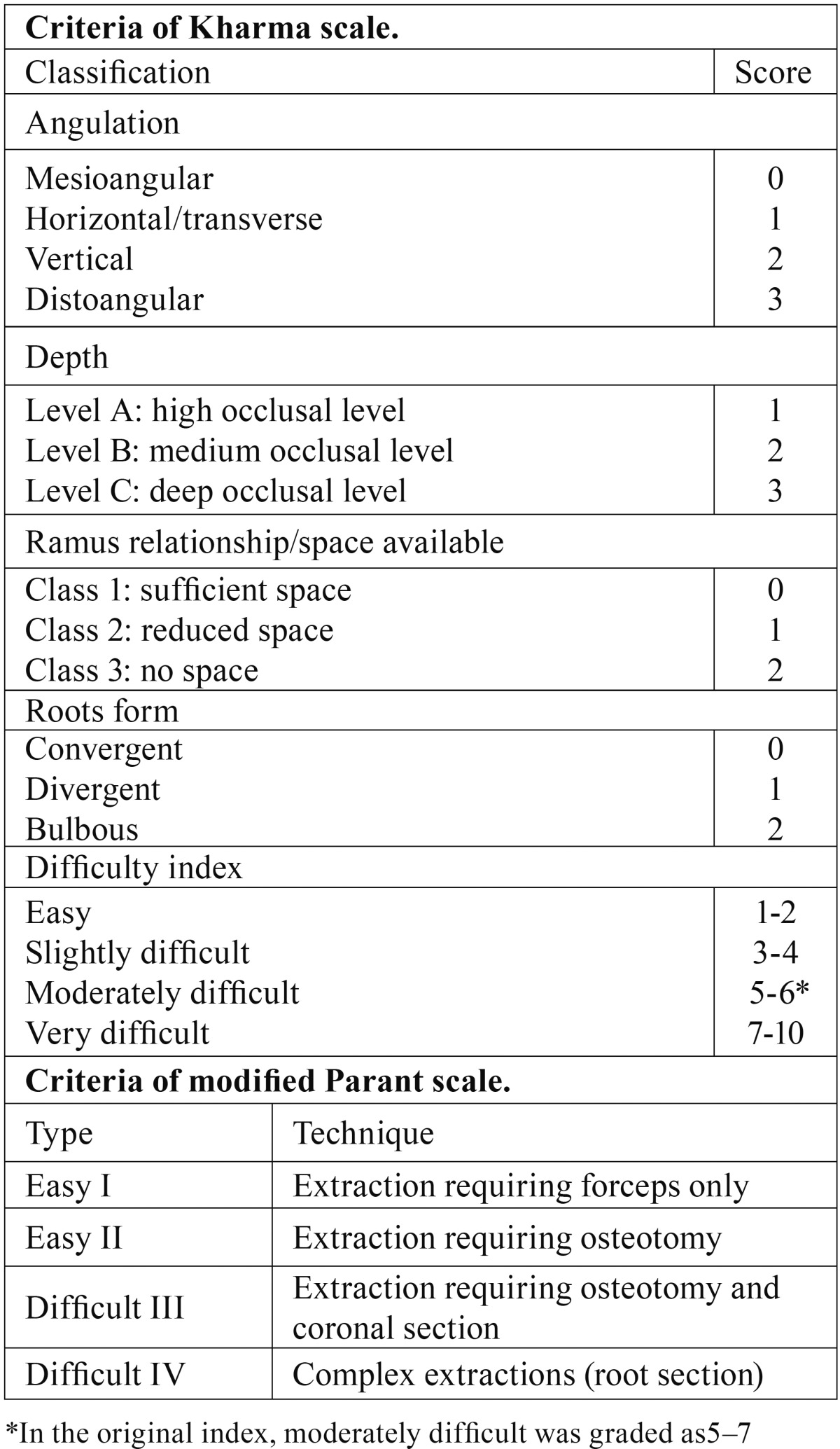
Criteria of Kharma and modified Parant scales.

By using descriptive statistics of IBM SPSS Statistics 23, sensitivity, specificity, and likelihood ratios were calculated considering the MPS as a reference. In addition, the correlation between the operative time and the difficulty of operation as proposed by both Kharma and modified Parant scales were also assessed by analysis of variance test considering a probability values less than 0.05 as significant.

## Results

 Table 2 review the classification of difficulty of 49 extractions by preoperative Kharma scale and postoperative MPS.

**Table 2:**
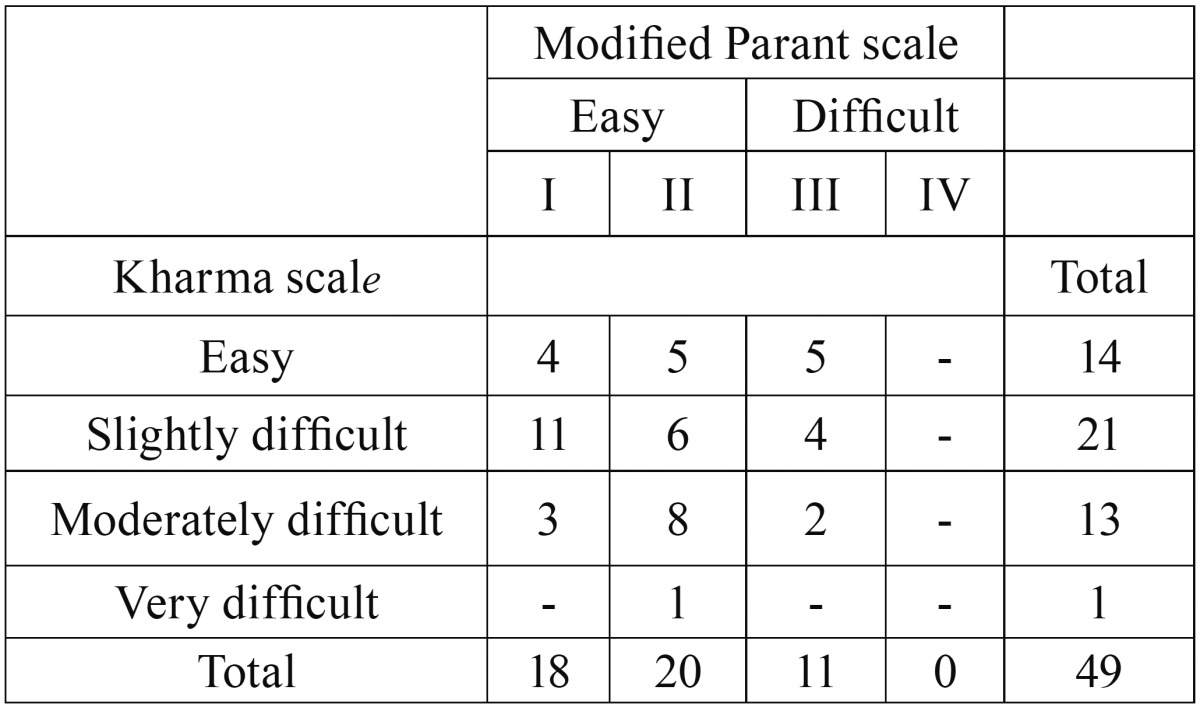
Classification of 49 extractions by preoperative Kharma scale and postoperative modified Parant scale.

The results indicate that 18 extractions are easy I according to MPS. By contrast, only 14 cases are classified as easy by Kharma scale. Kharma scale showed a low sensitivity (18.2%) for difficulty prediction of difficulty (11 extractions classified as difficult by the MPS, among them, only 2 cases were classified as moderately difficult by Kharma scale), and limited specificity of 68.4%.

Likelihood ratios for prediction of each class of Kharma scale were not significant as they ranged between 0.5 and 2 ( Table 3).

**Table 3:**
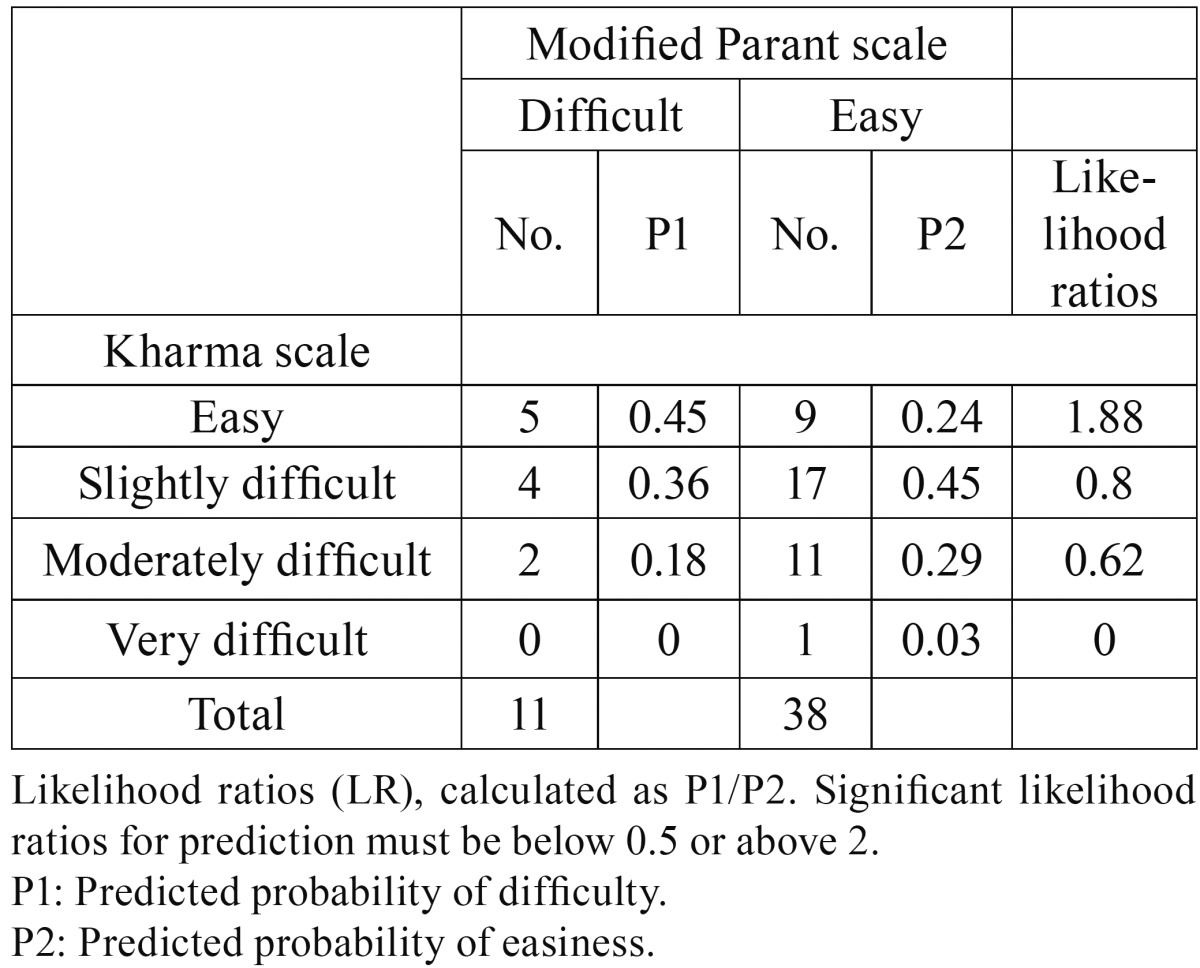
Predictive values (P1, P2) and likelihood ratios of the different Kharma categories for prediction of each modified Parant category.

The mean time of operative duration of each category of Kharma and modified Parant scale illustrated in  Table 4 which indicated that operations with longer duration was significantly correlated with higher modified Parant scores (*P*= .007). By contrast, no significant correlation was exist with Kharma scores (*P*= .716).

**Table 4:**
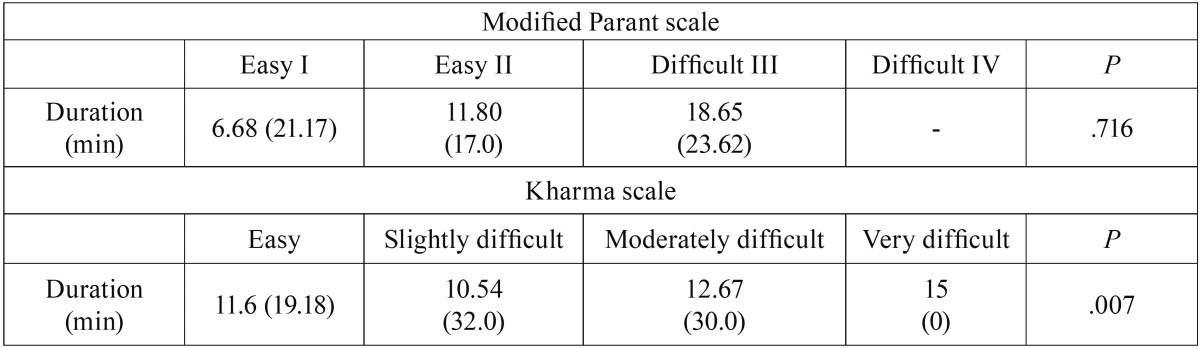
Mean (range) duration of operation (min) in each category of Kharma and modified Parant scale.

## Discussion

Clinical and radiographical findings should be considered preoperatively for correct evaluation of L3M extraction. It helps in prediction of operative difficulty for correct treatment plant and improves the level of patients’ satisfaction with the treatment received (1,8).

Several methods were established for preoperative estimation of difficulty like Pederson scale, which was used by clinicians as a useful tool to predict of L3M extraction difficulty (1). However, The meta-analysis of the current literatures concluded that Pederson scale is not a reliable prediction index in L3M surgery (9).

WHARFE scale (10) was also proposed, but is rarely used in practice duo to their complexity (1). Other variables had been considered in MRACBS scale (6) including L3M relation to inferior alveolar and lingual nerves. It is of limited clinical application due to the need to the cone beam computed tomography in classification.

Santamaria *et al.* (11) points to the importance root patterns in determining L3M extraction difficulty. Other researchers (2,3) took into account the curvature, width and number of L3M roots in their difficulty prediction index.

Kharma scale proposed a new difficulty prediction index based on 4 factors: tooth angulation, the depth of the third molar in the mandible, the relationship with the ramus/space available, and root form (7). It is similar to Pederson index in that it measures the same parameters in addition to root forms, and close to Yuasa scale (3) as the former assess the same parameters in addition to tooth angulation. However, Kharma scale, in this study, reported 85.7% false +ve. and 25.7% false –ve. and showed a very low sensitivity (18.2%) and a specificity of 68.4%. In contrast, Yuasa scale in a preliminary study (3) recorded 8.3% false +ve and 15% false –ve. which resulted in high sensitivity (85%) and specificity (92%). The false –ve cases causing problems for both the practitioners and patients (3).

In this study, the MPS was considered as a reference standard index of surgical difficulty as it found reliable and consistent with operative difficulty by researchers (1,7,8,12). The results of this study was in agreement with previous studies where the MPS was found significantly correlated with surgical time (*P*=0.007). However, Kharma scale fail to correlate with *P* value of 0 .716. The results indicate that the Kharma scale has poor sensitivity when over 85% of difficult extractions were not identified. In addition, likelihood ratios for prediction of the different difficulties of the Kharma scale from the categories of MPS also indicate that Kharma scale is of little value in predicting operative difficulty (a significant likelihood ratios for prediction must be below 0.5 or above 2) (13). This is may be owing to the lack of consideration of various relevant factors, such as bone density, periodontal space, flexibility of the cheek, and nerve proximity. In addition, curvature of the root is sometimes an unpredictable factor, as it is often not visible in panoramic radiographs (3).

In conclusion, and depending on the current results, the proposed Kharma scale was unreliable as preoperative predictor of the L3M extraction difficulty, and both radiological and clinical information must be taken into account.
